# Synthesis of Silver Nanoparticles From Cymodocea rotundata Leaf Extract and Their Biological Activities

**DOI:** 10.7759/cureus.49316

**Published:** 2023-11-23

**Authors:** T Darshinidevi, Vasugi Suresh, Pitchiah Sivaperumal, Elangovan Dilipan

**Affiliations:** 1 Physiology, Saveetha Dental College and Hospitals, Saveetha Institute of Medical and Technical Sciences, Saveetha University, Chennai, IND; 2 Prosthodontics, Saveetha Dental College and Hospitals, Saveetha Institute of Medical and Technical Sciences, Saveetha University, Chennai, IND

**Keywords:** nano silver, green synthesis of agnp, silver nitrate, silver ions, health care, anti-inflammatory, anti-oxidant, nanotechnology

## Abstract

Aim: Silver nanoparticles (AgNPs) are considered to be a very significant and intriguing type within the category of metallic nanoparticles, particularly in the context of their involvement in biological applications. The objective of this research is to use the green synthesis method in order to synthesize AgNPs by using the leaf extract of *C. rotundata*. Furthermore, the study aims to evaluate the antioxidant and anti-inflammatory properties of these nanoparticles.

Materials and methods: Fresh and healthy specimens of *C. rotundata* were gathered from Palk Bay, Tamil Nadu, India, and afterward subjected to a thorough washing process using tap water. The cleaned materials were air-dried and then fragmented into small bits and finely ground. The ethanolic extract of seagrass was then combined with a solution containing 1 millimolar (mM) silver nitrate (AgNo_3_). The decrease of silver ions in the solution was frequently measured using a UV-visible spectrophotometer. Synthesized AgNPs were investigated for antioxidants by DPPH (2,2-diphenyl-1-picrylhydrazyl) assay and anti-inflammatory activity was measured by protein-denaturation assay.

Results: The use of *C. rotundata* leaf extract in the green synthesis of AgNPs, in the presence of 1 mM AgNO_3_, led to a noticeable alteration in the colour of the mixture, transitioning from a pale hue to a brown shade. This change in colour serves as evidence of the reduction of AgNo_3_ ions to silver ions, thereby facilitating the creation of AgNPs. The duration of the bio-reduction process of silver ions in the reaction mixture was observed to be two hours. The antioxidant and anti-inflammatory activity showed promising activity for AgNPs.

Conclusion: This study concluded that *C. rotundata* had antioxidant capabilities, and AgNPs derived from *C. rotundata* have potential use in pharmaceuticals and medication administration.

## Introduction

The marine environment possesses extreme habitats characterized by significant fluctuations in pressure, salinity, temperature, and biological habitats, making it very intricate [1]. Recent studies have provided evidence about the pharmacological properties of chemicals obtained from marine sources, including their anti-cancer, anti-microbial, anti-fungal, and anti-inflammatory effects. Seagrasses, classified as submerged marine angiosperms, play a crucial role as primary producers throughout coastal oceans, excluding polar areas. *Cymodocea* species have been used for their calming properties in newborns, and their potential to alleviate discomfort during pregnancy and to treat anti-malarial activity such as potential larvicidal agent against *Aedes aegypti *mosquito larvae. Nanoparticles are characterized as particles with dimensions less than 100 nm, exhibiting distinct or enhanced properties in comparison to larger particles present in the bulk material. They exhibit distinctive characteristics with regard to their dimensions, distribution, and morphology [2].

The use of 'theranostic nanoparticles' encompasses a diverse array of applications within the diagnostic field, including optical imaging, magnetic resonance imaging (MRI), ultrasound, computed tomography, and nuclear imaging (including single-photon computed tomography and positron emission tomography). Numerous diverse types of nanoparticles, including gold, platinum, iron, and iron oxides, among others, have been recognized for their role in various therapeutic procedures. Metallic nanoparticles have shown significant practical promise and have seen rapid advancements, making them very viable for therapeutic applications, notably in the domains of diagnostics and imaging. Bio-synthesized silver nanoparticles (AgNPs) have drawn the interest of several research investigations due to their flexibility and variety of biological activities [3].

The optical phenomenon referred to as localized surface plasmon resonance (LSPR) enables a diverse range of applications for AgNPs, contingent upon their size and form [4]. The catalytic property of AgNPs is enhanced by their high surface area, which enables efficient binding and coordination of many elements [5]. However, the manufacturing techniques used for nanoparticles are accompanied by challenges, such as their limited stability [6].

Nanoparticle technologies have been used in the realm of therapy to address several medical conditions, including cancer, HIV/AIDS, ophthalmic and respiratory illnesses, as well as neurological disorders. Plant extracts are considered a viable option within the realm of biological approaches due to their advantageous characteristics, such as minimal maintenance needs, biosafety, widespread accessibility, and cost-effectiveness [7].

Using non-toxic solvents to make evenly distributed AgNPs that are biocompatible with host cells has proven to be a better way to make effective drug delivery systems than using chemicals. Additionally, it leads to a lower level of contamination and environmental damage in comparison. Previous research reported that AgNPs doped with seagrass extract showed promising activity anti-oxidant and anti-cancer activity [8]. The investigation on apoptosis also revealed that the biocompatibility levels of these AgNPs are greater compared to Cisplatin, a widely used chemotherapeutic agent. The research conducted and examined the efficacy of silver theranostic nanoparticles in combating human breast cancer cells and their potential to cause apoptosis [9]. Therefore, the primary objective of this work is to assess the process of green synthesis of AgNPs using an ethanolic extract derived from *Cymodocea rotundata*. Additionally, the study aims to assess the antioxidant and anti-inflammatory properties of the synthesized nanoparticles.

## Materials and methods

Sourcing of the sample

**Figure 1:**
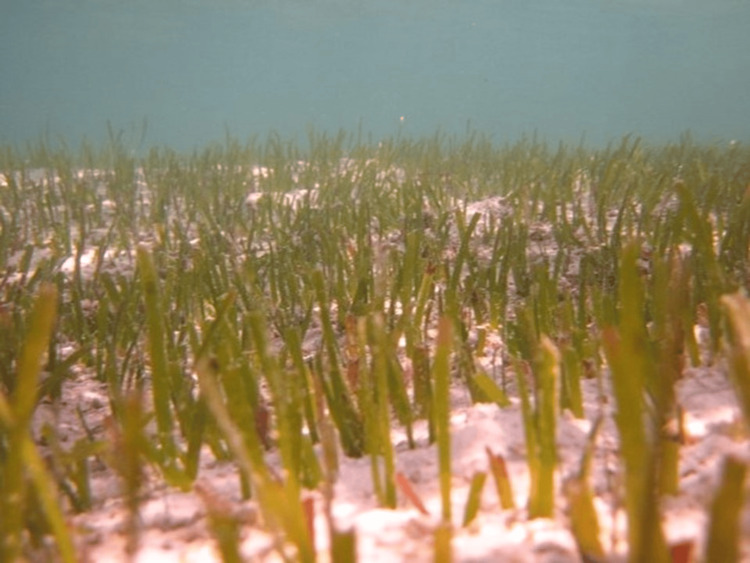
Seagrass sample, Cymodocea rotundata, collected from Palk Bay, Tamil Nadu, India

The bioactivity of several seagrass species was investigated for the production of nanoparticles. However, there is a limited amount of verified reports on species belonging to the *Cymodoacea* taxonomic group. Therefore, in this research endeavour, fresh, healthy *C. rotundata* sample (Figure 1) was collected from Palk Bay, Tamil Nadu, India, and washed in tap water to eliminate surface pollutants. Then the samples were transported to the laboratory and the experiment was carried out at the Department of Physiology, Saveetha Dental College and Hospitals, Chennai, India. After three weeks of shadow drying, the washed materials were chopped into small bits and coarsely pulverized using an electric blender. For future usage, powdered samples were sealed and kept out of the sun. 

Synthesis of silver nanoparticles

*C. rotundata* powder (10 gm) was combined with 100 millilitres of ethanol solution containing an 80% ethanol concentration. The resulting mixture was subjected to boiling for a duration of 30 minutes, followed by cooling and then filtration using Whatman filter paper no. 1. A 40-ml aqueous extract of *C. rotundata* was promptly introduced into a 60 ml solution containing 1 mM silver nitrate (AgNo_3_). The solution was subjected to a reduction of silver by placing it in an orbital shaker at room temperature. The reduction of silver ions in the solution was regularly monitored using a UV spectrophotometer. The acquired data included the optimal time needed for the reaction to reach completion. The AgNPs were purified using a series of centrifugation steps at a speed of 8000 rpm for a duration of 10 minutes. Subsequently, the resulting pellet was dispersed three times in deionized water in order to eliminate water-soluble biomolecules, such as proteins and secondary metabolites. The water-suspended nanoparticles were subjected to a 24-hour vacuum drying process in order to remove moisture from the nanoparticles.

Anti-oxidant activity

The α, α-diphenyl-β-picrylhydrazyl (DPPH) test was used in order to investigate the reducing capacity of antioxidants. The free radical known as DPPH, which is purple in colour, changes colour as it interacts with antioxidants, going from yellow to purple as a result. When determining the percentage of DPPH that each sample was able to scavenge, the absorbance at 520 nm was measured and tracked. The DPPH solution was made in ethanol and then combined with the test samples in a concentration of 20-100 µg/ml [10].

Anti-inflammatory activity

The anti-inflammatory effect of the AgNP extract was evaluated at various concentrations by dissolving it in a dimethyl sulfoxide (DMSO) solution. The AgNP extract was combined with a 1% aqueous solution of bovine serum albumin, and the pH was adjusted to 6.3 using 1N hydrochloric acid. Following the incubation and heating process, the absorbance at a wavelength of 660 nm was determined. The inhibition percentage was then computed using diclofenac sodium as the reference standard drug and DMSO as the control [11].

Statistical analysis

Analysis of variance (ANOVA) and Duncan's multiple comparison technique were used in order to determine the degree to which the samples differed from one another using IBM SPSS Statistics for Windows, Version 21.0 (Released 2012; IBM Corp., Armonk, New York, United States). When p < 0.05, it was considered that there was a significant difference.

## Results

**Figure 2:**
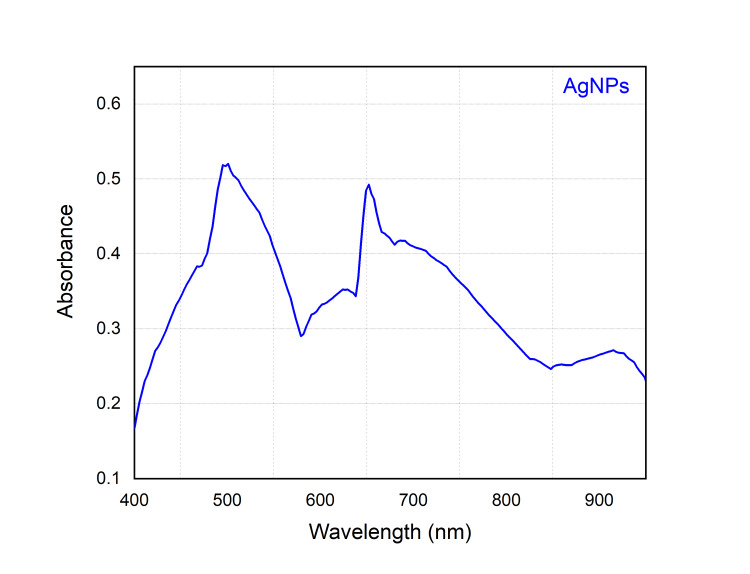
UV-vis absorption spectra of Cymodocea rotundata-mediated AgNPs (C. rotundata + AgNO3) AgNPs: silver nanoparticles; AgNO3: silver nitrate

The aqueous extract obtained from the leaves was mixed with a 1 mM solution of AgNo_3_ to generate an AgNo_3_ solution with a concentration of 25%. Due to the presence of phenolic and flavonoid compounds in the seagrass extract, a decrease in the concentration of Ag+ ions was what caused the observed transition to silver oxide (AgO) colouration. The observed change in colour of the solution, namely from light brown to dark brown, may be attributed mostly to the excitation of surface plasmon resonance (SPR) in AgNPs. Based on findings from previous research, it was observed that the seagrass-mediated silver nanoparticles exhibited a dark brown hue and had an absorbance peak spanning from 420 to 480 nanometers (Figure 2).

**Figure 3:**
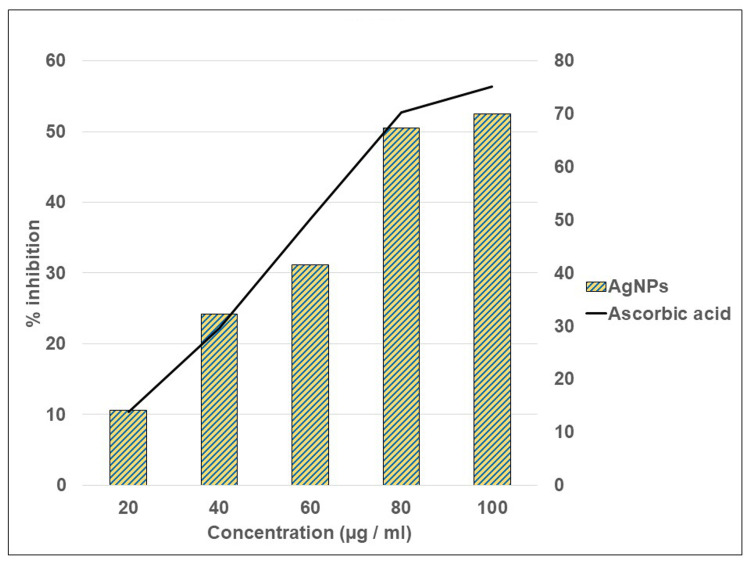
Antioxidant activities of AgNPs from Cymodocea rotundata AgNPs: silver nanoparticles

The results showed a positive relationship between the concentration of AgNPs made from seagrass extract and their antioxidant activity. The scavenging rate exhibited a range of values, spanning from 10.57% to 52.5%. This range was found to be comparable to that of ascorbic acid, which served as a reference compound. Additionally, the IC50 concentration of the nanoparticle was determined to be 72.17 µg/mL, as seen in Figure 3.

**Figure 4:**
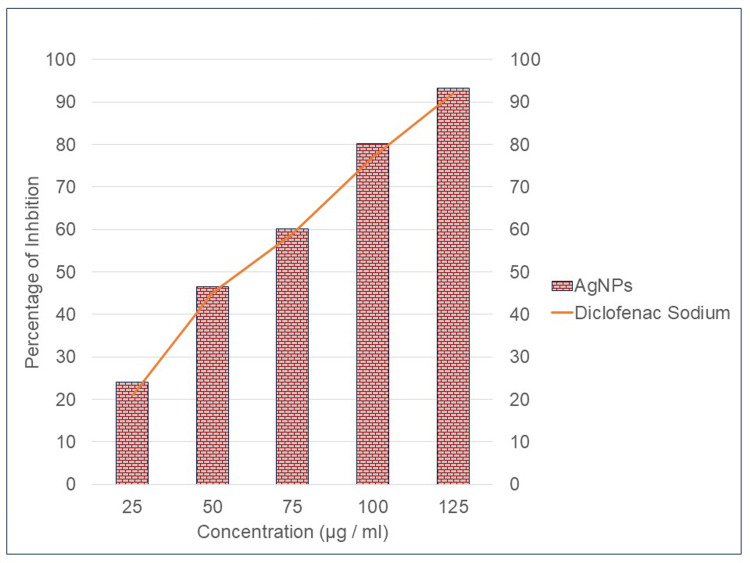
Anti-inflammatory activity of AgNPs from Cymodocea rotundata AgNPs: silver nanoparticles

The protein denaturation activity of the standard medication (diclofenac sodium) was determined to be 77.17%. With a score of 80.25 ± 1.77%, the AgNP demonstrated the highest level of anti-inflammatory action (Figure 4). The sample concentration and the standard concentration were both adjusted such that they were equal to 50 µg/ml in order to be normalised.

## Discussion

The addition of seagrass extract to a solution containing 1 mM of AgNO_3_ led to a noticeable alteration in the colour of the mixture, transitioning from a pale hue to a brown shade. This change in colour serves as evidence for the reduction of AgNO_3_ ions into silver ions, thereby facilitating the synthesis of AgNPs. The duration of the bio-reduction process of silver ions in the reaction mixture was observed to be two hours.

The use of biomaterials for the synthesis of AgNPs has garnered significant interest among researchers due to its expeditious, cost-effective, and ecologically sustainable protocol, which provides a streamlined approach to the biosynthesis process [12]. The nano-biotechnological capabilities of a number of biologically relevant materials with potential for use in medicine [13].

There is an increasing recognition of the significance of marine species in serving as a reservoir of unique materials. The ocean offers a considerable reservoir of unique compounds and has been identified as the most extensive unexplored source of natural molecules with potential medicinal properties. This is mostly due to the fact that marine organisms constitute over half of global biodiversity. Despite being a minor taxonomic group, seagrass is important economically because they are thought to be a storehouse of numerous bioactive substances that interact with inorganic and chemical nanoparticles. Early research claimed that the seagrass-AgNPs produced by *C. rotundata* had a dark-brown colour [14].

The observed change in colour of the reaction mixture upon being exposed to *C. rotundata* provides evidence supporting the formation of AgNPs via the reduction of aqueous Ag+ ions. The surface plasmon vibrations of the AgNPs were stimulated, resulting in the manifestation of a reddish-brown colouration in the material. The reaction mixture yielded AgNPs with an absorption peak of 420 nm. The broadening of the peak indicated the presence of polydisperse particles. The dimensions, morphology, and dielectric properties of the metal nanoparticles, as well as the dielectric properties of the surrounding medium, all have an impact on the frequency and breadth of the surface plasmon absorption [15]. The seagrass-AgNPs displayed the reported spherical form. Due to the presence of phytochemicals acting as stabilisers in the *C.* *rotundata* extract, field emission scanning electron microscopy (FESEM) examination verified the spherical shape of nanoparticles with low agglomeration [16].

Based on available research, it has been observed that the form of metal nanoparticles plays a crucial role in determining their optical and electrical characteristics, leading to large variations. The prominent, sharp signal seen at around 3 eV in the case of silver corresponds specifically to the absorption of crystalline AgNPs. The absorption of the amine group by the surface of the nanoparticle is evident, and it is mostly recognized as the principal agent responsible for the reduction of silver ions. The observed negative zeta potential of around 29.2 mV may be considered optimal for surface charge. The presence of high absolute zeta potential values and surface electrical charge on nanoparticles might impede agglomeration due to the presence of strong repulsive interactions between particles [16,17]. The cytotoxic effects of a methanolic extract of *C. rotundata* on HeLa cells also forecasted its potential in terms of tumour reduction [18].

The deadly effects of AgNPs are attributed to the active physicochemical interactions between silver atoms and the functional groups of intracellular proteins, as well as the nitrogen bases and phosphate groups present in DNA [19]. Additional investigation into the isolation and characterization of the cytotoxic constituent of the seagrass species *C. rotundata* has the potential to unveil previously undiscovered chemical compounds that might be of significant use in therapeutic settings. It is imperative to consider the *C. rotundata-*mediated AgNPs as a significant biocontrolling agent that warrants further investigation for its potential in the development of novel pharmaceuticals [20].

Limitations

It is vital to acknowledge that the current investigation focused on in vitro assessments. In order to enhance the credibility and reliability of the AgNP, it is essential to conduct in vivo research and clinical trials for the purpose of validating its effectiveness and safety.

## Conclusions

The marine angiosperm *C. rotundata*, which has therapeutic significance, was used in the synthesis of green AgNPs, as documented in current literature. The bioactive components of *C. rotundata* may include functional groups that might potentially participate in the reduction and stabilisation of AgNPs. The comparison between the produced AgNPs and the aqueous extract of *C. rotundata* demonstrated a considerable superiority of the former in terms of scavenging free radicals. The study revealed that *C. rotundata* had antioxidant capabilities, and AgNPs derived from *C. rotundata *have potential use in pharmaceuticals and medication delivery.
